# The Role of Glucagon-Like Peptide-1 Receptor Agonists in Post ST-Segment Elevation Myocardial Infarction Care: A Scoping Review

**DOI:** 10.1007/s40471-025-00375-5

**Published:** 2025-11-10

**Authors:** Leticia Alves Soares, Cynthia Paniagua, Julie Nguyen, Ajla Kojic, Elena Rose Sanfrey, Madison Emilee Reyome, Liliana Aguayo, Elisabeth Lilian Pia Sattler

**Affiliations:** 1 Department of Nutritional Sciences, College of Family and Consumer Sciences, University of Georgia, Athens, GA USA; 2 University of Georgia College of Pharmacy, Athens, GA USA; 3 Department of Clinical and Administrative Pharmacy, College of Pharmacy, University of Georgia, Athens, GA USA; 4 Nell Hodgson Woodruff School of Nursing, Emory University, Atlanta, GA USA

**Keywords:** GLP-1 receptor agonists, ST-elevation myocardial infarction, Cardioprotection, Exenatide, Liraglutide, Cardiovascular outcomes

## Abstract

**Purpose of review:**

This scoping review synthesizes current evidence on glucagon-like peptide-1 receptor agonist (GLP-1RA) use after ST-elevation myocardial infarction (STEMI), highlighting their potential as adjunctive therapy.

**Recent findings:**

Ten studies investigated exenatide and liraglutide in adults with STEMI, evaluating imaging, clinical, mechanistic, and safety outcomes. GLP-1RA use was safe and well tolerated. Exenatide demonstrated improvements in infarct size, myocardial salvage, and cardiac function, although two trials in broader STEMI populations reported no post-infarction improvements. Across three trials, liraglutide was associated with improved myocardial salvage, infarct size, left ventricular ejection fraction, stroke volume and no-reflow, supported by favorable biomarker changes, but without significant reductions in major cardiovascular events.

**Summary:**

While most existing evidence is based on studies with limited generalizability, GLP-1RA use shows promise in improving post-STEMI outcomes. The consistent benefits reported support the need for larger, multicenter trials to clarify GLP-1RA role in cardioprotection and long-term outcomes.

## Introduction

An ST-elevation myocardial infarction (STEMI) typically results from the complete occlusion of one or more coronary arteries and is characterized by (a) ST-segment elevation in at least two contiguous leads on an electrocardiogram (ECG), or by (b) a new left bundle branch block with ischemic repolarization patterns [1]. Because STEMI more often involves complete arterial occlusion, myocardial damage in STEMI is typically more extensive and transmural compared with Non-ST-elevation myocardial infarction (NSTEMI) [2], which contributes to higher rates of short-term mortality [3]. For example, in a multi-center observational study, STEMI patients had a higher in-hospital mortality rate (11.6% vs. 8.7%; *p* < 0.0001) and recurrent myocardial infarction (MI) (3.9% vs. 2.5%; *p* < 0.0001) than NSTEMI patients [3].

Although not used in acute treatment, it has been hypothesized that glucagon-like peptide-1 receptor agonists (GLP-1RA) can aid in heart failure (HF) management in post-MI patients by improving glucose uptake, promoting the release of guanosine monophosphate (cGMP) and cyclic adenosine monophosphate (cAMP), ultimately enhancing coronary flow and cardiac function [4]. Additionally, GLP-1RA agents may stimulate the growth of elastic fibers in cardiac tissue, contributing to favorable cardiac remodeling, a key factor in recovery following myocardial injury [4].

In vitro, GLP-1RA have shown to prevent cell death in heart cells (specifically HL-1 murine cardiomyocytes) [5]. Even in GLP-1 receptor knockout mice, GLP-1RA administration still provided cardioprotection, suggesting that some of its effects may occur independently of receptor binding [6]. In a canine model of ischemia-reperfusion, GLP-1RA infusion improved left ventricular function, accelerated recovery of regional motion (*p* < 0.05), and enhanced myocardial relaxation in treated dogs, supporting the potential protective role of GLP-1RA in facilitating recovery from myocardial stunning after reperfusion [7].

Considering their potential benefits, this review summarizes the current literature on the use of GLP-1RA in patients with STEMI, focusing on their role as an adjunctive therapy following the event. While a growing number of studies support the cardiovascular benefits of GLP-1RA, their specific role in post-STEMI management remains underexplored. By synthesizing available data on their cardioprotective effects in this high-risk population, this review aims to highlight their potential as a novel supportive strategy for improving post-STEMI outcomes.

## Methods

### Search Strategy and Eligibility Criteria

The PRISMA-ScR (Preferred Reporting Items for Systematic Reviews and Meta-Analyses extension for Scoping Reviews) guidelines were followed to ensure a systematic approach. Literature searches were conducted in PubMed and Web of Science from September 2024 to May 2025. Search terms combined GLP-1RA terms (GLP-1 receptor agonist, glucagon-like peptide-1, exenatide, liraglutide, semaglutide, dulaglutide, and albiglutide) with terms related to STEMI and cardiovascular outcomes (STEMI, ST-elevation myocardial infarction, acute myocardial infarction, AMI, infarct size, left ventricular function, cardiac remodeling, major adverse cardiovascular events, MACE, mortality, myocardial infarction, reperfusion injury, and cardioprotection). The search was limited to peer-reviewed studies in English and human participants.

To be included, studies were required to investigate effects of at least one GLP-1RA in adults with STEMI, report outcomes relevant to heart health (i.e., infarct size, left ventricular function, or major adverse cardiovascular events), and use either a clinical study (randomized controlled trials (RCTs), interventional studies, or post hoc analyses from RCTs) or observational study design using clinical data. Studies that focused only on blood sugar control, as well as reviews, commentaries, conference abstracts, or case reports, were excluded from this review.

## Study Selection, Data Extraction, and Data Synthesis

All references were imported into EndNote for duplicate removal. After de-duplication, the remaining records were exported to Microsoft Excel, where a single reviewer conducted the title and abstract screening and full-text eligibility assessment based on predefined inclusion and exclusion criteria. Full texts were reviewed when necessary. For each selected study, information was extracted on the study authors and year, design, population, type of GLP-1RA investigated and its use, outcomes measured, and main findings. Key limitations reported by the study authors were also recorded. The results were summarized descriptively, organized by the GLP-1RA evaluated and the type of cardiovascular outcome reported.

## Results

### Search Results

**Fig. 1:**
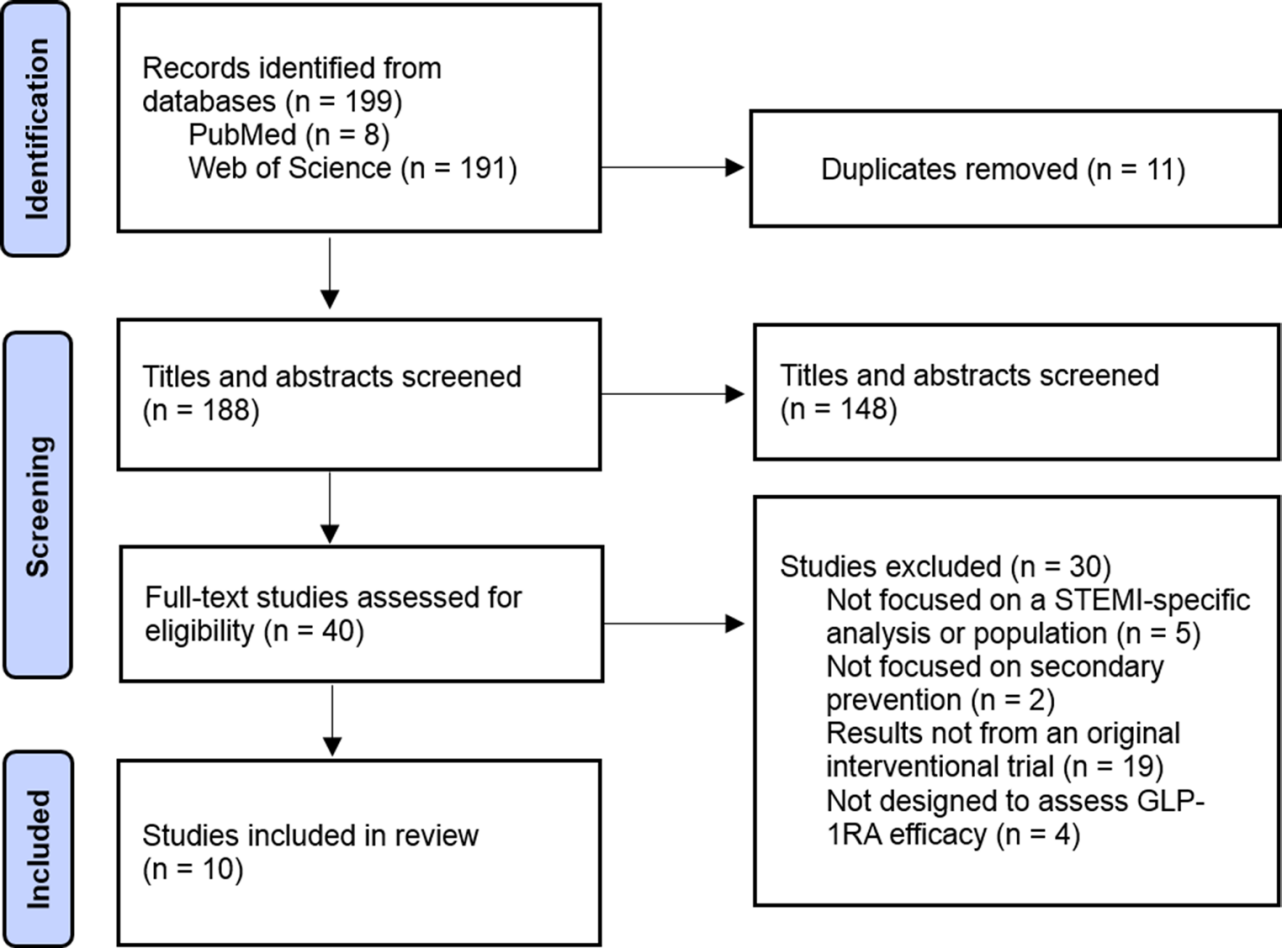
PRISMA 2020 flow diagram for study selection on GLP-1RA efficacy in STEMI populations

We identified 199 articles through database searches. After importing the records into EndNote, 11 duplicates were removed, leaving 188 articles for screening. The title and abstract screening process excluded 148 articles, which left a total of 40 full-text articles to be assessed for eligibility. Of these, 30 were excluded based on predefined criteria, resulting in 10 articles included in the final review (Fig. 1). No additional articles were identified through manual reference list searches.

Out of the 10 articles included, one was published in 2021 [8], while nine were published between 2012 and 2017 [9–17], as shown in Table 1. Eight studies were RCTs [8–10, 13, 14, 16, 17], two were post hoc analyses derived from RCTs [12, 15]. Of the GLP-1RA agent studied, seven articles evaluated exenatide [8, 10–15], followed by three that investigated liraglutide [9, 16, 17]. The outcomes assessed across the included studies varied, as demonstrated in Table 2, which summarizes the characteristics of studies assessing the role of GLP-1RA in STEMI. Overall, nine studies reported imaging-based measures, including infarct size, myocardial salvage, and left ventricular function [8–16]. Five articles evaluated clinical endpoints, including mortality and HF hospitalization [10, 11, 13–15]. Nine studies assessed biomarkers and mechanistic or procedural outcomes, such as cardiac injury markers, endothelial dysfunction biomarkers, no-reflow, and reperfusion injury [8–13–14, 16', 17] Four studies pre-specified safety and tolerability as endpoints [10, 13, 14, 16].

**Table 1:** Summary characteristics of peer-reviewed studies included

Study Characteristics	Number of Studies (%) *N* = 10
Publication Year	
2012–2017	9 (75%)
2021	1 (10%)
Study Design	
Randomized Controlled Trials (RCTs)	8 (80%)
Post Hoc Analyses from RCTs	2 (20%)
GLP-1 RA Studied	
Exenatide	7 (70%)
Liraglutide	3 (30%)
Outcomes Assessed	
Imaging-Based Measures	9 (90%)
Clinical Endpoints	5 (50%)
Biomarkers and mechanistic/procedural outcomes	9 (90%)
Safety and Tolerability	4 (40%)

**Table 2:** Characteristics of studies assessing the role of GLP1-RA in STEMI

Study	Study Design	Population (*n*)	Intervention	Primary and Secondary Endpoints	Major Findings	Safety/Adverse Events
Bernink FJP et al., 2012 [10]	Multicenter, double-blind, placebo-controlled RCT	39	IV exenatide just prior to PCI: 5 µg in 30 min, then 20 µg/24 × 72 h	MI size at 4 months; safety and feasibility, MACE, glucose regulation, Left ventricular function.	No MACE at 6 weeks or 4 months; no difference in glucose regulation, infarct size, or LVEF; trend toward smaller infarct size in TIMI 0/1 flow subgroup (35% vs. 47%, *p* = 0.094).	Nausea significantly higher with exenatide; no serious events
Lønborg J et al., 2012 [11]	Multicenter, double-blind, placebo-controlled RCT	105	IV exenatide 15 min prior to PCI: 0.12 µg/min in 15 min, then 0.043 µg/min × 6 h	Salvage index at 3 months; final infarct size, peak troponin T, left ventricular function, 30-day clinical events (death, MI, stent thrombosis, stroke)	Exenatide increased salvage index (0.71 vs. 0.62, *p* = 0.003) and reduced infarct size relative to AAR (0.30 vs. 0.39, *p* = 0.003); no effect on LVEF, troponin T, or 30-day clinical events.	Well tolerated; no adverse events
Roos ST et al., 2016 [13]	Multicenter, double-blind, placebo-controlled RCT	91	IV exenatide prior to PCI: 5 µg in 30 min, then 20 µg/day × 72 h	Final infarct size; myocardial salvage, left ventricular function, microvascular obstruction, MACE, biomarkers, and exenatide plasma levels	No difference in infarct size, LVEF, or MACE at 4 months	Nausea significantly higher with exenatide
García del Blanco et al., 2021 [8]	Multicenter, double-blind, placebo-controlled RCT	222	IV exenatide prior PCI: 0.12 µg in 15 min, then 0.043 µg/min × 6 h	Infarct size at 3–7 days; myocardial salvage index, transmurality index, left ventricular function, microvascular obstruction	No reduction in infarct size (%LV mass: 19.9 vs. 18.9, *p* = 0.72), myocardial salvage index, transmurality index, LVEF, or relative microvascular obstruction	Well tolerated, no adverse events.
Lønborg et al., 2012 [12]	Post hoc analysis of multicenter, double-blind, placebo-controlled RCT	327	IV exenatide 15 min prior to PCI: 0.12 µg/min in 15 min, then 0.043 µg/min × 6 h	Final infarct size at 3 months; Infarct size adjusted for area at risk; Salvage index; Peak troponin T; LVEF at 3 months	Exenatide reduced infarct size (8% vs. 11% LV, *p* = 0.015) and increased salvage index (0.75 vs. 0.66, *p* = 0.012); no effect on troponin T or LVEF; no benefit in long system delay.	No adverse events reported; treatment well tolerated
Kyhl et al., 2016 [15]	Post hoc analysis of Multicenter, double-blind, placebo-controlled RCT	334	IV exenatide 15 min prior to PCI: 0.12 µg/min in 15 min, then 0.043 µg/min × 6 h	Composite of all-cause mortality and admission for HF over median 5.2 years; individual component (all-cause mortality and HF)	No difference in composite outcome or mortality; fewer HF admissions with exenatide (11% vs. 20%, *p* = 0.042).	No serious adverse events; exenatide well tolerated
Woo et al., 2013 [14]	Single center, open label RCT	58	SC + IV exenatide: 20 µg in 5 min prior PCI, then 40 µg × 48 h	Infarct size; left ventricular function; C-reactive protein; NT-proBNP; safety/tolerability	Infarct size smaller in exenatide group (12.8 ± 11.7 g vs. 26.4 ± 11.6 g, *p* < 0.01; CK-MB/troponin release also reduced); improved left ventricular function; reduced hs-CRP; no effect on NT-proBNP.	Gastrointestinal side effects common in exenatide group; no serious adverse events
Chen WR et al., 2016 [9]	Single center, double-blind, placebo-controlled RCT	77	SC liraglutide: 1.8 mg in 30 min prior PCI, then 7-day taper (0.6, 1.2, 1.8 mg)	Salvage index at 3 months; infarct size; no-reflow; left ventricular function; biomarkers; MACE	Liraglutide increased salvage index at 3 months (0.66 vs. 0.53, *p* < 0.01); reduced final infarct size; improved LVEF; lower hs-CRP; no significant difference in MACE	No serious adverse events reported; liraglutide was well tolerated
Chen WR et al., 2016 [17]	Single center, double-blind, placebo-controlled RCT	196	SC liraglutide: 1.8 mg in 30 min prior PCI, then 7-day taper (0.6, 1.2, 1.8 mg)	Prevalence of no-reflow immediately after PCI; Biomarkers associated with cardiac injury, inflammation, oxidative stress, and vascular function	Liraglutide reduced no-reflow after PCI (5% vs. 15%, *p* = 0.01); reduced oxidative stress biomarkers; improved vascular function markers; no significant difference in short-term MACE	No serious adverse events reported; liraglutide was well tolerated
Chen WR et al., 2015 [16]	Single center, double-blind, placebo-controlled RCT	85	SC liraglutide: 1.8 mg in 30 min prior PCI, then 7-day taper (0.6, 1.2, 1.8 mg)	Left ventricular function at 3 months; no-reflow; Biomarkers associated with cardiac injury, inflammation, oxidative stress, and vascular function	Improvement in LVEF at 3 months + 4.1 (95% CI + 1.1% to + 6.9%) (*p* < 0.001); lower prevalence of no-reflow with liraglutide (7% vs. 18%, *p* < 0.05); reduced oxidative stress markers; improved vascular function markers	No serious adverse events reported; liraglutide was well tolerated

## Clinical Evidence of GLP-1RA in Acute STEMI: Agent-Specific Findings

### Exenatide

#### Study Populations and Designs

Overall, seven studies investigated the role of exenatide in patients with acute STEMI, including five RCTs [8, 10, 11, 13, 14] and two post hoc analyses [12, 15]. Sample sizes ranged from 39 to 334, and all patients were treated with primary percutaneous coronary intervention (PCI). Most studies focused on first-time STEMI [8, 10–15], with symptom onset within 6–12 h [8, 10–15], and Thrombolysis in MI (TIMI) flow grade 0–1 prior to intervention [8, 10–14]. While some trials applied strict criteria, such as excluding patients with prior MI, diabetes [11, 13], multivessel disease [12, 13], severe liver or kidney disease [10, 11] or impaired left ventricular function [14], others, particularly post hoc analyses, included broader patient populations to enhance statistical power [8, 12, 15]. All studies assessed the effects of exenatide compared to placebo, with slight variations in inclusion thresholds and angiographic findings. Across all studies, exenatide was compared to placebo as an adjunctive therapy to PCI, with the goal of reducing infarct size, improving myocardial salvage, preserving left ventricular function, or influencing long-term outcomes such as HF hospitalization. Six studies administered exenatide intravenously (IV) [8, 10–13, 15], and one study utilized a combination of subcutaneous (SC) and IV routes [14].

#### Intervention Approaches

From the studies assessed in this review, two trials employed an initial IV bolus of 5 µg over 30 min, followed by a continuous IV infusion at a rate of 0.84 µg/h or 20 µg per 24 h for up to 72 h to maintain therapeutic plasma concentrations during the critical post-reperfusion period [10, 13]. In studies aiming to target specific plasma levels (0.03–0.3 nmol/L), exenatide was infused at 0.12 µg/min for 15 min, then reduced to 0.043 µg/min for an additional 6 h [8, 11, 12, 15]. One study combined a 10 µg SC and 10 µg IV bolus given 5 min before reperfusion with twice-daily 10 µg SC injections over the following two days [14]. These protocols were designed based on prior safety data and pharmacokinetic modeling to optimize cardioprotection while avoiding adverse effects.

#### Main Findings

Results regarding cardioprotective effects of exenatide in patients with STEMI undergoing primary PCI were mixed. A few studies demonstrated significant cardioprotective benefits [11, 12, 14]. In these trials, exenatide was associated with approximately a 20%−50% reduction in infarct size (*p* = 0.003–0.015) [11, 12, 14], improved myocardial salvage index by 14–19% (*p* = 0.012–0.023), [11, 12] and lower infarct size-to-area-at-risk (AAR) ratios by approximately $\:\approx\:\:$18–31% (*p* = 0.003–0.024), with most pronounced benefits observed among patients with anterior infarcts [11, 12]. These effects were particularly notable in patients treated early, with short system delays (total time from first medical contact to reperfusion of 132 min or less) [12]. Biomarkers of myocardial injury, creatine kinase–myocardial band (CK-MB) and troponin I, were significantly reduced in one study, and infarct mass was nearly half in the intervention group (12.8 ± 11.7 g vs. 26.4 ± 11.6 g; *p* < 0.01) [14]. Functional measures, such as left ventricular ejection fraction (LVEF) did not differ significantly in most studies, [8, 11, 13, 15] though one trial reported higher LVEF and improved diastolic function at six months with exenatide [14].

In contrast, two RCTs, including 91 [13] and 222 [8] patients, found no significant differences in infarct size, myocardial salvage index, cardiac biomarkers or LVEF between patients treated with exenatide and placebo groups. Final infarct size as a percentage of left ventricular mass ranged from 13% to 25% across these trials, with p-values consistently above 0.05 [8, 13]. Importantly, both trials observed very low major adverse cardiac event (MACE) rates, likely due to contemporary PCI procedures [8, 13]. A post hoc study found no reduction in the composite endpoint of mortality and HF hospitalization (24% vs. 27%; HR 0.80, *p* = 0.44), but did report a significant decrease in HF admissions alone (11% vs. 20%; HR 0.53, *p* = 0.042), suggesting a potential benefit for long-term cardiac remodeling [15]. In addition, the EXAMI study showed that high-dose exenatide was feasible and well-tolerated, with a non-significant trend toward reduced infarct size in patients with complete or near-complete blockage of the coronary artery (TIMI 0 to 1 flow) [10]. Across most studies included, gastrointestinal side effects like nausea were commonly reported, but usually manageable, and no major safety concerns were reported [10, 12–15].

### Liraglutide

#### Study Populations and Designs

Three RCTs conducted between 2015 [16] and 2016 [9, 17] by the same research group examined the cardioprotective effects of liraglutide in patients with acute STEMI (Table 2). All studies were single-center, double-blind, placebo-controlled trials conducted at the People’s Liberation Army (PLA) General Hospital in Beijing, China [9, 16, 17].

The sample size across the RCTs ranged from 77 to 196 participants. Inclusion criteria were consistent in all studies, requiring a diagnosis of STEMI within 12 h of symptom onset, chest pain lasting more than 30 min, ST-segment elevation of ≥ 0.1 mV in at least two contiguous ECG leads, and elevated troponin T levels [9, 16, 17]. Similarly, individuals who were unconscious at presentation or had severe conditions, including cardiogenic shock, hypoglycemia, diabetic ketoacidosis, renal insufficiency, or a history of MI, stent thrombosis, or coronary artery bypass grafting, were excluded from all studies [9, 16, 17]. The primary focus of each study differed to explore complementary aspects of liraglutide’s cardioprotective potential, while maintaining a consistent treatment protocol. The first study emphasized left ventricular functional recovery over three months using echocardiographic assessment [16]. The second trial targeted the occurrence of no-reflow as an early procedural endpoint, [17] while the third study evaluated myocardial salvage using advanced cardiac imaging [9].

#### Intervention Approaches

Liraglutide was administered via subcutaneous injection and standardized dosing regimen in all three studies. An initial dose of 1.8 mg was administered 30 min prior to primary PCI, typically while the patient was in the ambulance. This was followed by a tapered 7-day post-PCI regimen, consisting of 0.6 mg daily for 2 days, 1.2 mg daily for the subsequent 2 days, and 1.8 mg daily for the final 3 days. A matching placebo was administered on the same schedule as liraglutide in all studies, and patients from both groups (intervention and control) received traditional STEMI therapy, including aspirin, clopidogrel, statins, β-blockers, ACE inhibitors, and drug-eluting stent implantation during PCI [9, 16, 17].

#### Main Findings

Across all three RCTs, liraglutide demonstrated favorable effects on cardiac function, inflammatory profiles, and markers of myocardial injury in patients with STEMI undergoing primary PCI. Although there are differences in study focus, the results were mostly consistent in supporting liraglutide’s cardioprotective potential. All studies reported a significant improvement in LVEF in the liraglutide groups compared to placebo at three months, with increases of approximately 4 to 6% higher than in the placebo group (*p* < 0.01 in all trials) [9, 16, 17], with greater gains in patients with baseline LVEF below 50% [16]. Furthermore, treatment with liraglutide showed a significant 6.8 mL increase in stroke volume compared with controls [16].

Regarding inflammation and endothelial function, all trials showed consistent reductions in high-sensitivity C-reactive protein (hs-CRP), and increases in nitric oxide and nitric oxide synthase levels in the liraglutide groups [9, 16, 17]. Lower troponin and creatin kinase (CK) levels were also detected in two studies [9, 16, 17]. Regarding MACE, liraglutide groups showed a numerically lower incidence in one trial, but the difference was not statistically significant [9, 16, 17].

Despite these shared outcomes, each study had a distinct primary endpoint, finding a significantly higher salvage index in the liraglutide group and a smaller final infarct size, both in grams and as a percentage of left ventricular mass [9], in the no-reflow study significantly lower incidence of no-flow in the liraglutide group (5%) compared to placebo (15%,) was observed (*p* = 0.01), alongside higher rates of TIMI flow grade 3 and myocardial blush grade 3 [17]. In the study evaluating left ventricular function, a lower incidence of no-reflow in the liraglutide group (7% vs. 15%) was also reported; however, the difference was not statistically significant, leading the authors to suggest that liraglutide may delay rather than prevent this phenomenon [16].

In the context of safety and tolerability, liraglutide was well tolerated across all studies. Mild and manageable adverse effects such as nausea and hypoglycemia were slightly more frequent in the liraglutide groups [9, 16, 17].

## Discussion

In this scoping review, we aimed to evaluate the cardiovascular effects of GLP-1RA in adults with STEMI. Although evidence remains limited, the available studies suggest that GLP-1RA is safe in this context and may provide cardioprotective benefits as adjunctive therapy.

Among the agents studied, exenatide has shown to be safe and, although mild nausea was reported, usage was overall deemed well-tolerated, even at high doses. In fact, GLP-1RA demonstrated promising cardiovascular effects, including reductions in infarct size and improvements in myocardial salvage, and cardiac function, [11, 12, 14] particularly in patients with shorter system delays and total ischemic times, highlighting the potential importance of early exenatide administration in maximizing therapeutic benefit [12]. This aligns with findings from a preclinical study, which also observed a time-dependent component to the cardioprotective effects of exenatide [18].

A non-significant trend toward reduced infarct size was observed in patients with complete or near-complete coronary occlusion [10]. Additionally, treatment with exenatide was associated with a significant reduction in hospital admissions for HF, but this benefit did not translate into a reduction in the composite endpoint of all-cause mortality and HF hospitalization, as mortality rates were slightly higher in the exenatide group [15]. The ambiguity of these findings may indicate that while exenatide exerts beneficial effects on cardiac remodeling and short-term HF management, these improvements may not translate into improved overall survival.

Despite previously reported benefits of exenatide in STEMI, two multicenter trials did not observe significant improvements in post-MI outcomes [8, 13]. All studies of exenatide in acute STEMI, administered the drug before or around the time of primary PCI, aiming to target the period most vulnerable to ischemia-reperfusion injury [10–12, 14, 15]. However, unlike studies with favorable outcomes, these two studies did not report detailed timing of exenatide administration or system delays, and total ischemic times may have been longer or more variable, which could diminish the drug’s efficacy during a critical window of ischemia-reperfusion. Additionally, one study enrolled patients with heterogeneous characteristics representative of real-world practice [8], whereas earlier studies applied strict selection criteria, including shorter ischemic time and anterior infarcts, which may have increased the chance of observing a treatment effect.

Overall, these findings suggest that exenatide may confer cardioprotective effects under specific conditions, particularly when administered early in treatment. Nevertheless, most trials faced methodological and design challenges, including small sample sizes, limited statistical power [8, 10, 11, 13–15], and high exclusion rates [10, 11, 13] which may affect the reliability and generalizability of these results, underscoring the importance of larger, and rigorously designed studies to confirm these findings and to establish the optimal timing and patient population for exenatide therapy in acute STEMI management [19]. If validated in larger trials, these benefits may translate into improved patient outcomes and support exenatide as a promising adjunctive strategy in the treatment of STEMI.

In the context of liraglutide, all three trials conducted by Chen et al. consistently enrolled patients with STEMI undergoing emergency PCI, exploring variations in sample size, exclusion criteria, and primary endpoints [9, 16, 17]. Regardless of these differences, liraglutide consistently improved measures of cardiac recovery, including myocardial salvage, infarct size, and LVEF [9, 16, 17], with one trial demonstrating reduced no-reflow [16]. This consistent pattern of benefit across studies strengthens the hypothesis that liraglutide may have a cardioprotective effect in the acute STEMI setting. By enhancing systolic function and maintaining forward flow, it may contribute to improved functional recovery and help prevent adverse remodeling.

Beyond imaging and functional endpoints, the consistent biomarker findings across the trials provide important foundation about how liraglutide might exert its cardioprotective effects. Lower troponin and CK levels align with reduced myocardial damage. Decreases in endothelial and oxidative stress markers, such as hs-CRP and endothelin-1 and increases in nitric oxide and nitric oxide synthase levels [9, 16, 17] suggest anti-inflammatory and vascular protective effects. These mechanisms may also explain the lower rates of no-reflow and improved microvascular perfusion observed.

The studies included for liraglutide shared common limitations, including small sample sizes, single-center designs, and short treatment durations, which reduced statistical power and raised concerns about representativeness [9, 16, 17]. Further, key differences in study design precluded us from conducting a meta-analysis. Addressing these methodological issues will require larger, multicenter trials with methodological consistence, and longer follow-up to assess outcomes such as ventricular remodeling and HF. Nevertheless, the consistent pattern of evidence observed across trials suggests that liraglutide’s benefits extend beyond glucose control, reflecting a broader cardioprotective action that may support cardiac function and recovery after STEMI.

## Conclusions

In summary, GLP-1RA, specifically exenatide and liraglutide, demonstrate benefits that extend beyond glucose control, with consistent evidence of cardioprotective effects following STEMI. Most studies associated GLP-1RA use with improvements in myocardial salvage, infarct size, ventricular function, stroke volume, and microvascular perfusion. However, studies including broader STEMI populations did not confirm these effects, likely reflecting longer ischemic times, greater heterogeneity, and lower event rates for the outcomes measured. Overall, current evidence is promising but not yet conclusive, highlighting the need for larger multicenter studies to better establish their definitive role in cardiovascular care.

## Data Availability

No datasets were generated or analysed during the current study.

## Key References

- Garcia Del Blanco B, Otaegui I, Rodriguez-Palomares JF, Bayes-Genis A, Fernandez Nofrerias E, Vilalta Del Olmo V, et al. Effect of COMBinAtion therapy with remote ischemic conditioning and exenatide on the Myocardial Infarct size: a two-by-two factorial randomized trial (COMBAT-MI). Basic Res Cardiol. 2021;116(1):4. doi: 10.1007/s00395-021-00842-2.
  - This is the most recent study included (2021), combining exenatide and ischemic conditioning. Highlights both current evidence and limitations.
- Chen WR, Chen YD, Tian F, Yang N, Cheng LQ, Hu SY, et al. Effects of Liraglutide on Reperfusion Injury in Patients With ST-Segment-Elevation Myocardial Infarction. Circ Cardiovasc Imaging. 2016;9(12). doi: 10.1161/CIRCIMAGING.116.005146.
  - One of the most comprehensive and mechanistically focused trials showing liraglutide’s effect on reperfusion injury. Central to your scoping review.
- Lonborg J, Vejlstrup N, Kelbaek H, Botker HE, Kim WY, Mathiasen AB, et al. Exenatide reduces reperfusion injury in patients with ST-segment elevation myocardial infarction. Eur Heart J. 2012;33(12):1491-9. doi: 10.1093/eurheartj/ehr309.
  - Early and influential trial suggesting cardioprotective potential of exenatide in STEMI patients.
- Chen WR, Tian F, Chen YD, Wang J, Yang JJ, Wang ZF, et al. Effects of liraglutide on no-reflow in patients with acute ST-segment elevation myocardial infarction. Int J Cardiol. 2016;208:109-14. doi: 10.1016/j.ijcard.2015.12.009.
  - This larger trial addressed the clinically relevant outcome of no-reflow, adding weight to the evidence for liraglutide’s cardioprotective role in STEMI.
